# Influence of Temperature Variation on the Vibrational Characteristics of Fused Silica Cylindrical Resonators for Coriolis Vibratory Gyroscopes

**DOI:** 10.3390/s20041032

**Published:** 2020-02-14

**Authors:** Pengbo Xiao, Zhinan Qiu, Yiming Luo, Yao Pan, Tianliang Qu, Kaiyong Yang, Hui Luo, Shiqiao Qin

**Affiliations:** College of Advanced Interdisciplinary Studies, National University of Defense Technology, Changsha 410073, China; xiaopengbo09@nudt.edu.cn (P.X.); qiuzhinan17@nudt.edu.cn (Z.Q.); luoyiming11@nudt.edu.cn (Y.L.); qutianliang@nudt.edu.cn (T.Q.); yky208@nudt.edu.cn (K.Y.); luohui.luo@163.com (H.L.)

**Keywords:** fused silica cylindrical resonator, frequency mismatch, Q factor, temperature, coriolis vibratory gyroscope

## Abstract

The fused silica cylindrical resonator is a type of axisymmetric resonator that can be used for Coriolis vibratory gyroscopes. Although the resonant frequency, frequency mismatch, and Q factor are natural properties of the resonator, they can change with temperature. Therefore, the temperature drift severely limits the detection accuracy and bias stability of the gyroscope. In this paper, the influence of temperature variation on the vibrational characteristics of fused silica cylindrical resonators was investigated. Experiments were performed on a fused silica cylindrical resonator coated with Cr/Au films. It was shown that at the temperature range from 253.15 K to 353.15 K, the resonant frequency linearly increased with temperature, the frequency mismatch remained unchanged, and the Q factor gradually increased till about 333.15 K, when it began to decrease. Meanwhile, the change of thermoelastic damping with temperature may dominate the variation of Q factor at the temperature range from 253.15 K to 353.15 K. This phenomenon was theoretically analyzed and the variation trends of results were consistent with the theoretical analysis. This study indicates that, for the fused silica cylindrical resonator, to discover the influence of temperature variation on the resonant frequency, frequency mismatch, and Q factor, there are certain rules to follow and repeat. The relationship between temperature and frequency can be established, which provides the feasibility of using self-calibration based on temperature characteristics of the resonator for temperature drift compensations. Additionally, there is an optimum temperature that may improve the performance of the Coriolis vibratory gyroscope with the fused silica cylindrical resonator.

## 1. Introduction

Coriolis vibratory gyroscopes (CVGs), especially those with axisymmetric shell resonators, have many advantages including high reliability, considerable stability, long life, low power consumption, and a maintenance-free concept, and are widely used in navigation fields and platform stabilization systems [1,2,3,4,5,6,7,8,9]. For this type of gyroscope, there are mainly two types of axisymmetric shell resonators—hemispherical shell resonators and cylindrical shell resonators [10]. The representative products using hemispherical shell resonators include the Northrop Grumman H130 series and the Safran Regys series [11,12]. The Northrop Grumman Hubble hemispherical resonator gyroscope (HRG), in particular, has been reported to have bias stability of 0.00008°/h and angle random walk (ARW) of 0.00001°/h^1/2^ [8], and the Safran Regys 20 has been reported to have bias stability better than 0.01°/h [13]. The representative products using cylindrical shell resonators include the InnaLabs Inc. INL-CVG series; the INL-CVG-GU200, in particular, has been reported to have bias stability of 0.03°/h and ARW of 0.004°/h^1/2^ [14]. The University of Michigan has reported a micro birdbath shell resonator gyroscope with bias stability of 0.0103°/h [15]. The fused silica cylindrical shell resonators, in particular, are a type of high-performance resonators with high Q factor, which have a simpler structure and are much easier to manufacture compared with hemispherical shell resonators. Therefore, they have broad applications in the tactical-level inertial navigation system [16]. Our research group has reported fused silica cylindrical resonators with the Q factor approaching 10^6^ in 2016 [17] and 3 × 10^6^ in 2019 [18].

For CVGs, the temperature effect is the main reason causing the variation of the output in addition to the input angular velocity [19,20]. The crucial vibrational characteristics of the resonator that limit the overall performance of the gyroscope, including the resonant frequency, frequency mismatch, and Q factor, are all temperature-dependent [21]. Investigating the variation of vibrational characteristics with temperature is the first step for studying the temperature-drift compensations of CVGs. To give a few examples, Li et al. theoretically studied the output of hemispherical resonator gyroscope (HRG) under temperature influence, they obtained the frequency splitting formula and that the HRG has maximum disturbance limit in stable operation section [22]. Tang et al. discussed the static temperature characteristics of MEMS gyroscopes and derived that zero bias of the gyroscope is dependent on temperature and temperature gradient [23]. Therefore, gyros have better performance in environments with stable temperatures. Shao et al. experimentally investigated the resonant frequency and Q factor of microscale hemispherical shell resonators under temperature influence. A linear and positive temperature coefficient of frequency was found, and the Q factor showed an inverse trend at temperatures above 40 °C [24]. Wu et al. investigated the temperature characteristics of vibration mode axes for metal cylindrical resonators. They found that a small frequency split and low thermal elastic modulus coefficient are necessary for the cylinder resonator [20]. Therefore, the influence of temperature variation on the resonant frequency and the Q factor, there may be certain rules to follow. Wang et al. set up a mathematical model of the relationship between the temperature and the natural frequency of HRG, and compensations were made for the output drift of HRG based on natural frequency through a stepwise linear regression method [19], which shows the feasibility of making compensations based on the resonant frequency since the external temperature sensors may cause delays. Zega et al. investigated the frequency stability and thermoelastic effects for slotted tuning fork MEMS resonators (SETF). They theoretically computed the natural frequency and the quality factor of a MEMS resonator under different levels of material doping and different material orientations to obtain the analytical model of an idealized SETF. Meanwhile, they introduced slots in the resonator beams and applied an optimization tool to realize temperature stability together with a high quality factor [25]. Therefore, there are several means, like changing the levels of material doping, and changing the material orientation, which are helpful to mitigate the thermal drift of natural frequency of silicon MEMS resonators. Additionally, a careful design can help reduce the thermal drift even when slots are inserted in the devices in order to decrease thermoelastic losses. Our group has previously reported experimental results on the temperature–frequency characteristics of fused silica cylindrical resonators, and the linear relationship between temperature and resonant frequency was demonstrated [16], but the experimental results of other vibrational characteristics variations, for example, Q factor and frequency mismatch variations, have not been reported. This paper intends to report the experimental results of the changes of resonant frequency, frequency mismatch, and Q factor of fused silica cylindrical resonators with temperature, and provide theoretical analysis on these changes, showing the feasibility of using self-calibration based on temperature characteristics of the resonator to obtain the real-time temperature for compensations and providing an optimum temperature for performance improvement.

This paper comprises five sections. Using electrostatic excitation and detection, methods to measure the vibrational characteristics of fused silica cylindrical resonators with different temperature are described in Section 2. The influence of temperature variation on the vibrational characteristics of fused silica cylindrical resonators was theoretically analyzed in Section 3.1, while numerical calculations of resonant frequency, frequency mismatch, and damping loss with different temperatures are presented in Section 3.2. Experimental results and discussions are presented in Section 4. Section 5 concludes this paper with a summary of the results.

## 2. Experiments and Methods

**Figure 1:**
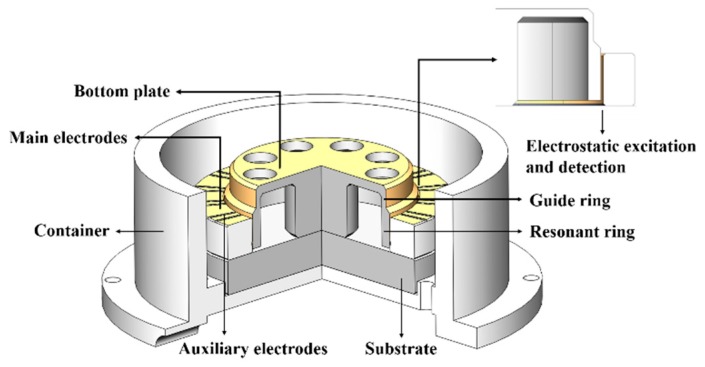
The sectional view of the resonator with electrodes.

In this research, a fused silica cylindrical resonator with a high Q factor was fabricated in the same way as stated in [17]. The sectional view of the resonator with electrodes is shown in Figure 1. The resonator has three main components, the resonant ring, the guide ring, and the bottom plate. The resonant ring has the maximum amplitude and is used for electrostatic excitation and detection, the guide ring is used for vibration transfer, and the bottom plate is used for support. The outer surface of the resonator was coated with Cr/Au (~20/60 nm) film by magnetron sputtering and the resonator was rigidly connected with a fused silica base through its supporting rod. A fused silica cylindrical ring with laser-cut electrodes was attached on the base outside the resonator with a gap of approximately 16 μm. The main electrodes were used to excite or detect resonator vibration, while the auxiliary electrodes were grounded to reduce signal interference. The assembly was vacuum packaged with a pressure of 10^−4^ Pa with getter. During the experiment, the whole package was placed in a temperature-controlled chamber.

### 2.1. Electrostatic Excitation and Detection

**Figure 2:**
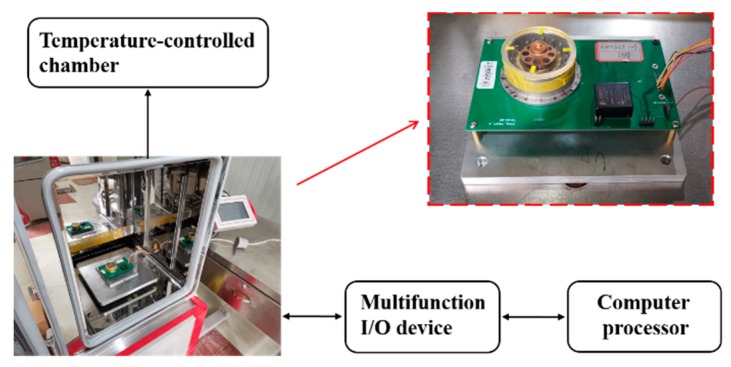
The diagram of the experimental setup used for the testing.

The outside metal film of the resonator and the main electrodes formed parallel plate capacitors used to excite or detect the vibration of the resonator from different directions. The constructions of electrostatic excitation and detection for fused silica resonators have been described in detail in [26,27]. Using a multifunctional I/O device, the actuating capacitors were connected to applied voltage sources. Excitation signals were generated by the multifunction I/O device with the controlled program designed and operated in the LabVIEW software, and all the relative parameters could be easily adjusted according to the actual conditions. Detection signals from sensing capacitors were collected by the multifunctional I/O device and processed by the LabVIEW program, as shown in Figure 2.

### 2.2. Measurement of Vibrational Characteristics

The temperature range was set from 253.15 K to 353.15 K with a 10 K step. Each time the temperature was adjusted, we waited about four hours for the resonator to reach a new equilibrium, then performed measurements of the vibrational characteristics. The cylindrical shell resonator works at *n* = 2 resonant mode. Two principle axes have their own resonant frequencies *f*1 and *f*2, and they are equal, ideally. However, the shell thickness and surface state of the machine-made resonators are imperfect, which results in mass and stiffness distribution variations and causes differences between *f*1 and *f*2. Supposing *f*1 > *f*2, therefore, the resonator has a pair of principal axes (the high-frequency principal axis, *f*1 and the low-frequency principal axis, *f*2), and the frequency split is defined as *f*1–*f*2, which is one of the main error sources in vibratory gyroscopes [28,29].

**Figure 3:**
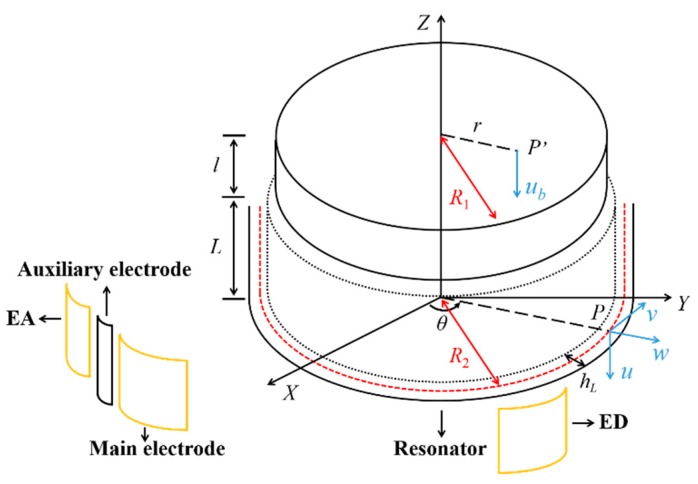
The structure diagram of the resonator with electrodes.

For the measurement of frequency and frequency mismatch, a pair of electrodes EA along with the low-frequency principal axis was used for actuation, while the pair ED in quadrature with EA was used for detection, as shown in Figure 3, and the structure shown has been modified for illustration purposes. A phase-locked loop (PLL) was applied to the detection signal. Meanwhile, an automatic gain control (AGC) loop was applied to realize the amplitude-control loop function, which maintained the amplitude constant. All control loops had already been programmed in the LabVIEW program previously mentioned. Therefore, the resonant frequency of the resonator was collected directly from the reference-phase loop. For the measurement of Q factor, the ring-down method was employed. After the vibration was stabilized by the PLL and AGC loop, the actuation signal was cut off and the ring-down time constant τ was recorded. The relation between Q factor and ring-down time constant is Q=πfτ [30], where *f* is the resonant frequency of the resonator. The measurement for the resonant frequency was repeated for the high-frequency principal axis, hence the frequency mismatch was acquired.

## 3. Theoretical Analysis

The intrinsic mode in the form of a four-node standing wave is investigated in this paper, the vibration of a cylindrical shell resonator can be expressed according to [31,32]

(1) $$\begin{array}{l} u=U(x)\cdot \cos(2\theta )\cdot \cos(\omega t)\\ v=V(x)\cdot \sin(2\theta )\cdot \cos(\omega t)\\ w=W(x)\cdot \cos(2\theta )\cdot \cos(\omega t)\\ u_{b}=U_{b}(r)\cdot \cos(2\theta )\cdot \cos(\omega t) \end{array}$$

where *u*, *v*, and *w* are respectively the tangential, axial, and radial displacement vector of a point *P* on the shell’s middle surface with the angular position θ, and *u_b_* is the displacement vector of a point $P^{\prime }$ on the bottom plate with the radial position *r*, as shown in Figure 3. U(x), V(x), W(x) and $U_{b}(r)$ are mode functions, which are presented in [33].

### 3.1. Theoretical Analysis

Since the stiffness of the resonator varies with the elastic modulus under varying temperatures, the resonant frequency of the resonator will also change. Assuming that the temperature-dependent elastic modulus of the resonator is [34,35,36,37,38]

(2) $$E(T)=E_{0}+k_{ET}E_{0}(T-T_{0})$$

where *E*(*T*), *E*_0_ are respectively the elastic modulus of the resonator at temperature *T* and *T*_0,_ *k_ET_* is the temperature coefficient for elastic modulus. Since the stiffness of the resonator is directly proportional to the elastic modulus, the stiffness variation induced by elastic modulus variation is

(3) $$\Delta K_{E}=k_{ET}K_{0}(T-T_{0})$$

where *K*_0_ is the stiffness at temperature *T*_0_. Considering the influence of thermal stress, the stiffness of the resonator becomes

(4) $$K=(K_{0}+\Delta K_{E})(1+\alpha \Delta T)=K_{0}(1+\frac{E(T)-E_{0}}{E_{0}})(1+\alpha \Delta T)$$

where α is the thermal expansion coefficient and $\Delta T=T-T_{0}$. Therefore, the resonant frequency of the resonator at temperature *T* is

(5) $$f_{T}=\sqrt{\frac{K}{m}}=\sqrt{\frac{K_{0}(1+\frac{E(T)-E_{0}}{E_{0}})(1+\alpha \Delta T)}{m}}=f_{0}\sqrt{\frac{E(T)(1+\alpha \Delta T)}{E_{0}}}$$

Substituting (2) into (5) and performing Taylor expansion on x(ΔT), $f_{T}$ becomes

(6) $$\begin{array}{l} f_{T}=f_{0}\sqrt{\frac{\left[E_{0}+k_{ET}E_{0}(T-T_{0})](1+\alpha \Delta T\right)}{E_{0}}}=f_{0}\sqrt{\left[1+k_{ET}\Delta T](1+\alpha \Delta T\right)}\\ =f_{0}{\left[1+x(\Delta T)\right]}^{1/2}=\{1+\frac{1}{2}x(\Delta T)-\frac{1}{8}x^{2}(\Delta T)+o[x^{2}(\Delta T)]\} \end{array}$$

where $x(\Delta T)=\alpha k_{ET}\Delta T^{2}+(\alpha +k_{ET})\Delta T$. Assuming the order of α and $k_{ET}$ are respectively about 10^−7^ and 10^−4^, and ΔT≤100 K, $f_{T}$ approximates to

(7) $$\begin{array}{l} f_{T}=f_{0}\{1+\frac{1}{2}[\alpha k_{ET}\Delta T^{2}+(\alpha +k_{ET})\Delta T]-\frac{1}{8}{\left[\alpha k_{ET}\Delta T^{2}+(\alpha +k_{ET})\Delta T\right]}^{2}\}\\ =f_{0}[1+\frac{1}{2}(\alpha +k_{ET})\Delta T] \end{array}$$

which indicates that the resonant frequency of the resonator linearly changes with temperature in this case. Meanwhile, assuming the resonant frequency excited in the low-frequency principal axis and the high-frequency principal axis at temperature *T*_0_ are respectively $f_{01}$ and $f_{02}$ with an initial frequency mismatch of $\Delta f=f_{02}-f_{01}$, then the frequency mismatch $\Delta f_{T}=f_{T02}-f_{T01}$ at temperature *T* equals to

(8) $$\Delta f_{T}=(f_{02}-f_{01})[1+\frac{1}{2}(\alpha +k_{ET})\Delta T]=f_{02}-f_{01}$$

Therefore, the frequency mismatch of the resonator remains a constant in this case.

The total loss of a cylindrical shell resonator mainly consists of five types: air-damping correlated 1/*Q_gas_*, support-loss correlated 1/*Q_sup_*, surface-loss correlated 1/*Q_sur_*, thermoelastic-damping correlated 1/*Q_thr_*, and internal-friction correlated 1/*Q_fri_*, as described by the following Equation (10),

(9) $$\frac{1}{Q}=\frac{1}{Q_{air}}+\frac{1}{Q_{sup}}+\frac{1}{Q_{sur}}+\frac{1}{Q_{thr}}+\frac{1}{Q_{fri}}$$

Since the fused silica cylindrical resonator was sealed in a vacuum packing container with a pressure of 10^−4^ Pa and the resonator was rigidly connected with the fused silica base, we neglected the air damping and the support loss in this research.

The resonator has a damaged layer on its surface due to machining and polishing. Such a damaged layer consists of the residual carbon of diamond paste and a hundred micron-thick layer of dislocations. It has inhomogeneous heat during vibration and leads to surface loss. According to the surface loss of the cylinder described in [39], the surface defect loss correlated 1/*Q_sur_* of the cylindrical resonator approximates to

(10) $$\frac{1}{Q_{sur}}=2h_{dam}\cdot (\frac{1}{L}+\frac{1}{l}+\frac{1}{R_{1}}+\frac{1}{R_{2}})\cdot \frac{ET\alpha^{2}}{c}\cdot \frac{2\pi f_{T}t}{1+{\left(2\pi f_{T}t\right)}^{2}}$$

where *L*, *l* are respectively the height of the resonant ring and the guide ring of the resonator, *R*_1_ and *R*_2_ are respectively the radiuses of middle surfaces of the guide ring and the resonant ring, *h_dam_* is the depth of the damaged layer, *E* is the elastic modulus, and α is the thermal expansion coefficient. The characteristic time *t* is introduced by Zener [40] as “relaxation time” to describe anelasticity in solids, and is derived as $t=\psi^{2}\frac{c}{\kappa }$, where ψ is the typical size of heat distribution [39], κ is the thermal conductivity coefficient, and *c* is the heat capacity.

The thermoelastic damping is a type of internal friction and the dissipation mechanism results from thermal conduction due to the temperature gradient in the fused silica. According to the thermoelastic damping of the cylindrical shell given by [40,41,42], the thermoelastic damping correlated 1/*Q_thr_*_1_ of the resonant ring is

(11) 1Qthr1=2|Im(ωmn)Re(ωmn)|=2|tanΞ2|

where Re(ωmn)=1R2EρψcosΞ2, Im(ωmn)=1R2EρψsinΞ2, ρ is the density, and Ξ=arctan(Im(ΔΩ2mn)Re(ΔΩ2mn)+Ω2mn). The parameters Im(ΔΩ2mn), $Re(\Delta \Omega^{2}{}_{mn})$, and $\Omega^{2}{}_{mn}$ are calculated in [41]. Similarly, the thermoelastic damping correlated 1/*Q*’*_thr_*_1_ of the guide ring is obtained, and the thermoelastic-damping correlated 1/*Q_thr_*_2_ of the bottom plate is given by [43],

(12) $$\frac{1}{Q_{thr2}}=D[\frac{6}{\delta^{2}}-\frac{6}{\delta^{3}}(\frac{\sinh\delta +\sin\delta }{\cosh\delta +\cos\delta })]$$

where $D=\frac{(1+\mu )E\alpha^{2}T}{(1-2\mu )\rho c}$, $\delta =\frac{h_{b}{}^{3/2}}{2R_{1}}{\left(\frac{10.21c}{\kappa }\right)}^{1/2}{\left[\frac{E\rho }{3(1-\mu^{2})}\right]}^{1/4}$, μ is the Poisson’s ratio, and *h_b_* is the thickness of the bottom plate. Therefore, the thermoelastic-damping correlated 1/*Q_thr_* of the resonator equals to 1/*Q_thr_*_1_ + 1/*Q*’*_thr_*_1_ + 1/*Q_thr_*_2_.

The internal friction is an energy loss in virtue of imperfect elastic stress, which is rendered irrecoverable, or unavailable for the resonator vibration. The internal-friction correlated 1/*Q_fri_* of the resonator is

(13) $$\frac{1}{Q_{fri}}=\frac{U_{fri_{1}}+U_{fri_{2}}+U_{fri_{3}}}{2\pi S}$$

where *S* is the potential energy of the resonator and the explicit expression of *S* is described in [33,44], $U_{fri_{1}}$, $U_{fri_{2}}$, and $U_{fri_{3}}$ are respectively the internal-friction loss of the radial, axial, and bottom plate of the resonator, and their explicit expressions are given according to the internal friction of cupped resonator described in [33]

(14) $$\begin{array}{l} U_{fri_{1}}=\frac{1}{24}\frac{E\xi h_{L}{}^{3}}{R_{2}{}^{3}}\int_{0}^{L}\int_{0}^{2\pi }\left(\frac{\partial^{2}v}{\partial \theta \partial t}-\frac{\partial^{3}w}{\partial^{2}\theta \partial t})\cdot (\frac{\partial v}{\partial \theta }-\frac{\partial^{2}w}{\partial^{2}\theta }\right)d\theta dx\\ +\frac{1}{24}\frac{E\xi h_{l}{}^{3}}{R_{1}{}^{3}}\int_{L}^{L+l}\int_{0}^{2\pi }\left(\frac{\partial^{2}v}{\partial \theta \partial t}-\frac{\partial^{3}w}{\partial^{2}\theta \partial t})\cdot (\frac{\partial v}{\partial \theta }-\frac{\partial^{2}w}{\partial^{2}\theta }\right)d\theta dx\\ U_{fri_{2}}=\frac{1}{24}ER_{2}\xi h_{L}{}^{3}\int_{0}^{L}\int_{0}^{2\pi }k_{x}\cdot \frac{dk_{x}}{dt}d\theta dx+\frac{1}{24}ER_{1}\xi h_{l}{}^{3}\int_{L}^{L+l}\int_{0}^{2\pi }k_{x}\cdot \frac{dk_{x}}{dt}d\theta dx\\ U_{fri_{3}}=\frac{1}{24}E\xi h_{b}{}^{3}\int_{r_{0}}^{R_{1}}\int_{0}^{2\pi }k_{y}\cdot \frac{dk_{y}}{dt}\cdot rd\theta dr \end{array}$$

where ξ is the internal friction coefficient of the material, *h_L_* and *h_l_* are respectively the thickness of the resonant ring and the guide ring, $k_{x}=\frac{W(x)''\cos2\theta \cos(\omega t)}{{\left\{1+{\left[W(x)'\cos2\theta \right]}^{2}\right\}}^{3/2}}$, and $k_{y}=\frac{U_{b}(r)''\cos2\theta \cos(\omega t)}{{\left\{1+{\left[U_{b}(r)'\cos2\theta \right]}^{2}\right\}}^{3/2}}$.

### 3.2. Numerical Calculations

**Figure 4:**
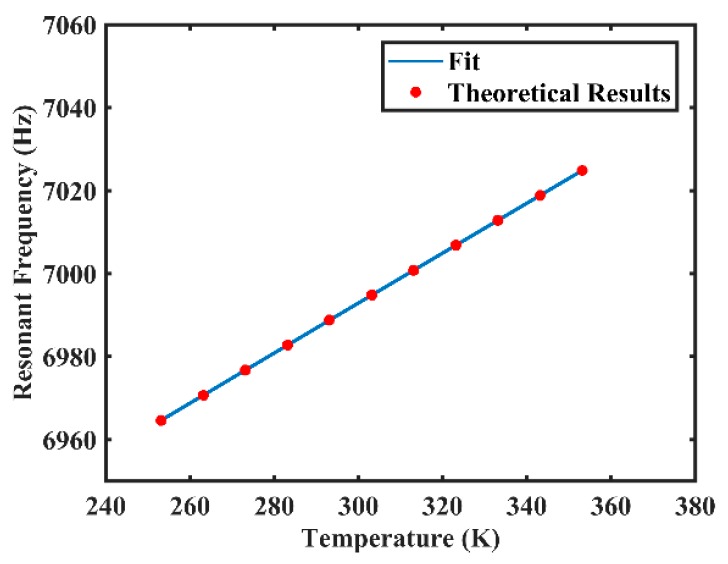
The fitted value of resonant frequency varying with temperature.

Table 1 presents some mechanical and thermal properties of fused silica used for the following numerical calculations, and the effects of temperature on elastic modulus, *E*, and Poisson’s ratio, μ, of fused silica are depicted in [36,37]. At the temperature range from 253.15 K to 353.15 K, the elastic modulus and the Poisson’s ratio both vary linearly with temperature as in $E(T)=1.267\times 10^{7}T+6.995\times 10^{10}$ and $\mu (T)=1\times 10^{-4}T+0.14$ [36,37], and other properties were considered as constants in this study. Table 2 presents the geometry parameters of the resonator. With these parameters, we can calculate the changes of specific vibrational characteristics with temperature according to the theoretical analysis in Section 3.1. According to Equation (5), numerical results show that the fitted value of resonant frequency is f=0.6021T+6812.2, and the goodness of fit of the fitting curve is 1, as shown in Figure 4. Meanwhile, according to Equation (7), the approximation of resonant frequency is f=0.6036T+6811.8 and the relative difference of the gradient is 0.25%, which indicates the reliability of the approximation.

**Figure 5:**
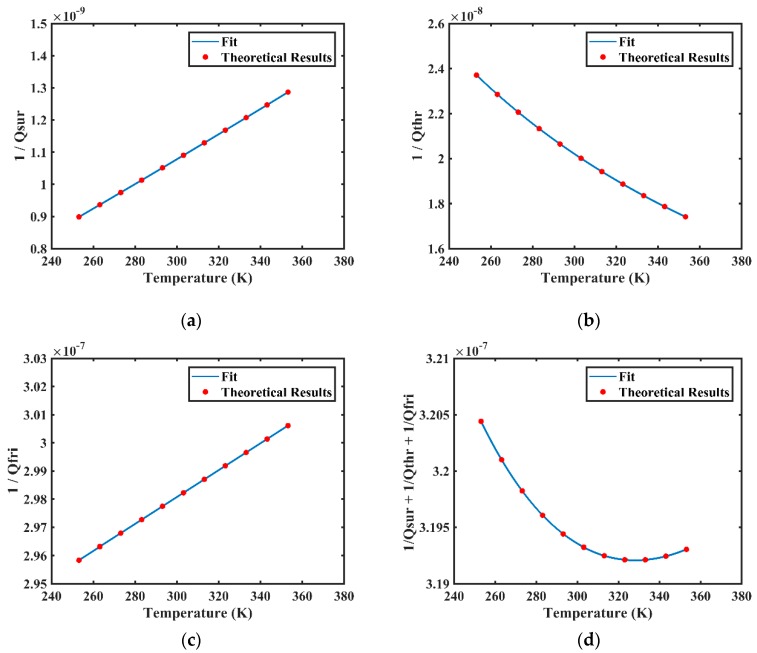
Numerical calculations of different types of damping loss varying with temperature at the temperature range from 253.15 K to 353.15 K: (**a**) the surface-loss correlated 1/*Q_sur_* varies with temperature; (**b**) the thermoelastic-damping correlated 1/*Q_thr_* varies with temperature; (**c**) the internal-friction correlated 1/*Q_fri_* varies with temperature; (**d**) the sum of 1/*Q_sur_*, 1/*Q_thr_*, and 1/*Q_fri_* varies with temperature.

Different types of damping, varying with temperature at the temperature range from 253.15 K to 353.15 K, were calculated and results are shown in Figure 5. According to Equation (10), the numerical calculation of surface-loss correlated 1/*Q_sur_* varying with temperature is shown in Figure 5a. The surface loss increases linearly from about 0.9 × 10^−9^ to 1.3 × 10^−9^ (increased by 44.44%) at the temperature range from 253.15 K to 353.15 K. According to Equations (11) and (12), the numerical calculation of thermoelastic-damping correlated 1/*Q_thr_* varying with temperature is shown in Figure 5b. The thermoelastic damping gradually decreases from about 2.38 × 10^−8^ to 1.72 × 10^−8^ (decreased by 27.73%) at the temperature range from 253.15 K to 353.15 K. According to Equations (13) and (14), the numerical calculation of internal-friction correlated 1/*Q_fri_* varying with temperature is shown in Figure 5c. The internal friction increases linearly from about 2.96 × 10^−7^ to 3.01 × 10^−7^ (increased by 1.66%) at the temperature range from 253.15 K to 353.15 K. Putting these three types of damping loss together, the numerical calculation of 1/*Q_sur_* + 1/*Q_thr_* + 1/*Q_fri_* varying with temperature is shown in Figure 5d. The total loss gradually decreases from about 3.204 × 10^−7^ to 3.192 × 10^−7^ till about 328 K when it begins to rise and reaches 3.193 × 10^−7^ at 353.15 K.

Therefore, at the temperature range from 253.15 K to 353.15 K, the resonant frequency increases linearly, the frequency mismatch remains unchanged, and the total damping loss gradually decreases till about 328 K when it begins to rise.

## 4. Experimental Results and Discussion

**Figure 6:**
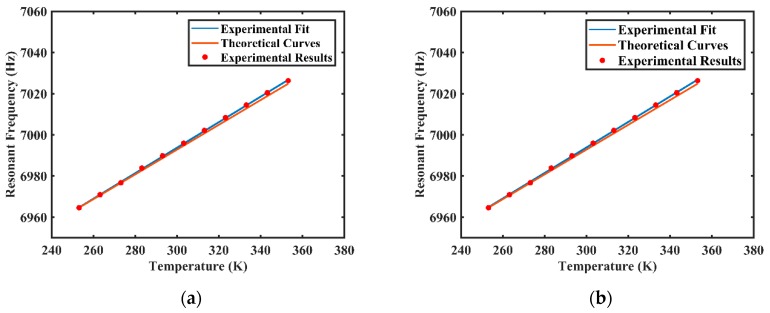
The experimental results of resonant frequency varying with temperature at the temperature range from 253.15 K to 353.15 K: (**a**) results from the heating process; (**b**) results from the cooling process.

The resonant frequency of the resonator excited in the low-frequency principal axis was measured during both the heating process and the cooling process—results are shown in Figure 6. Figure 6a shows that the fitted value of experimental resonant frequency in the heating process is $f_{1}=0.6196T+6807.9$, and the goodness of fit of the fitting curve is 0.9998. Figure 6b shows that the fitted value of experimental resonant frequency in the cooling process is $f_{1}=0.6193T+6808.1$, and the goodness of fit of the fitting curve is 0.9997. The error of experimental results between two processes may result from the accuracy level of the temperature-controlled chamber, about ±0.1 K, which may cause a frequency fluctuation of 0.12 Hz. Compared with the theoretical result f=0.6021T+6812.2, the relative differences of the gradient in the heating process and the cooling process are respectively 2.82% and 2.78%, which shows good consistency and repeatability. The error between experimental and theoretical results may result from the accuracy of mechanical and thermal properties used for theoretical calculations, for example, the elastic modulus, *E* has been approximated to linear change at the temperature range from 253.15 K to 353.15 K. Therefore, the resonant frequency is positively correlated with temperature and changes linearly at the temperature range from 253.15 K to 353.15 K. Considering that each temperature only corresponds to one resonant frequency, the resonant frequency of the resonator can be used as a real-time measure of temperature variation to compensate for the performance of CVG with the temperature-dependent gyro drift and the temperature-dependent noise. In addition, the experimental results of the frequency mismatch at different temperatures remain at 0.056 Hz, which are also consistent with theoretical analysis.

**Figure 7:**
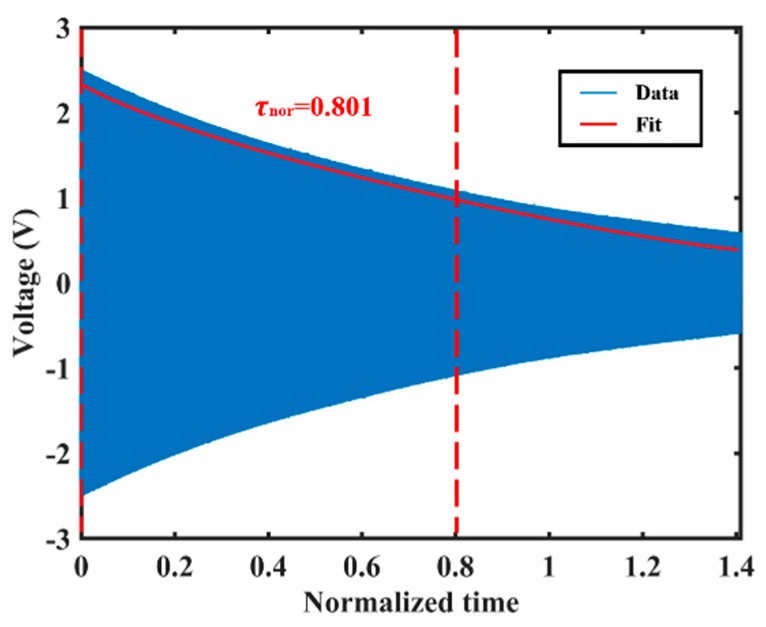
The measured normalized ring-down time constant of the resonator in 253.15 K.

The ring-down time constant measurement of the resonator in 253.15 K is shown in Figure 7, and the result is normalized with respect to the ring-down time constant at 333.15 K. It is shown that the normalized ring-down time constant (low-frequency axis excited) $\tau_{nor}$ of the resonator at 253.15 K is about 0.801.

**Figure 8:**
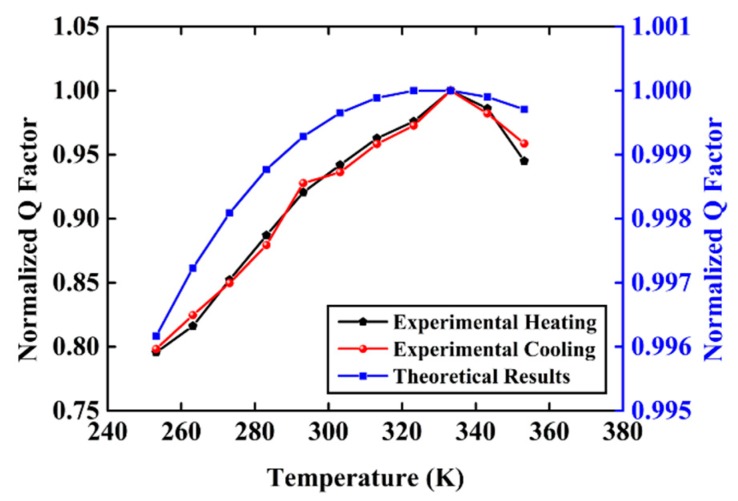
The measured and theoretical normalized Q factor varying with temperature at the temperature range from 253.15 K to 353.15 K.

The measurement for the normalized ring-down time constant was repeated for different temperatures, hence the Q factor of the resonator varying with temperature is acquired and the results are normalized with respect to the Q factor at 333.15 K, as shown in Figure 8. The normalized Q factor of the resonator in the heating process and the cooling process both show an upward trend from 253.15 K to about 333.15 K when it reaches the top, and it gradually decreases till 353.15 K, which shows good repeatability. The inverse of theoretical 1/*Q_sur_* + 1/*Q_thr_* + 1/*Q_fri_* in Figure 5d is also normalized with respect to the data at 333.15 K and displayed in Figure 8, which shows nearly the same trend and variation rate with the experimental curves. The error may result from the air damping, support loss, and other losses that have been neglected—although they are tiny and hardly vary with temperature, these losses still influence the exact value of Q factor. Therefore, there should be an optimal temperature for minimum damping loss, and the resonator may reach the highest Q factor at a certain temperature, which are consistent with the theoretical calculations. Meanwhile, according to the variation rate of different types of damping loss between two temperatures, the change of thermoelastic damping with temperature may dominate the variation of Q factor at the temperature range from 253.15 K to 353.15 K.

## 5. Conclusions

This paper reports the experimental results of the changes in resonant frequency, frequency mismatch, and Q factor under temperature influence. Experiments were performed on a film-coated fused quartz cylindrical resonator with ring electrodes. The resonant frequency increases linearly, while the frequency mismatch remains unchanged with temperature at the temperature range from 253.15 K to 353.15 K. Meanwhile, the Q factor of the resonator gradually increases from 253.15 K till 333.15 K, when it shows a downward trend, and the Q factor may reach its maximum at a temperature between 323.15 K and 343.15 K. Meanwhile, the change of thermoelastic damping with temperature may dominate the variation of Q factor at the temperature range from 253.15 K to 353.15 K. All the vibrational characteristics were measured both in the heating process and the cooling process, and the results showed good repeatability. These changes were theoretically analyzed by introducing the effects of temperature on some mechanical and thermal properties of fused silica, for example, elastic modulus and Poisson’s ratio into the resonant frequency and damping loss equations of the cylindrical resonator, and variation trends in experimental results were consistent with the theoretical analysis. Therefore, the relationship between temperature and resonant frequency is established, the resonant frequency of the resonator can be used as a real-time measure of temperature variation to compensate for the performance of a CVG with the temperature-dependent gyro drift and the temperature-dependent noise. In addition, there is an optimum temperature that may improve the performance of a CVG with the fused silica cylindrical resonator.

## Figures and Tables

**Table 1:** Mechanical and thermal properties of fused silica.

Component	Value	Units
Density, ρ	2.203 × 10^3^	Kg/m^3^
Damaged layer depth, *h_dam_*	100	μm
Heat capacity, *c*	772	JKg^−1^ K^−1^
Thermal conductivity, κ	1.39	W/mK^−1^
Thermal expansion coefficient, α	5 × 10^−7^	K^−1^
Typical size of heat distribution, ψ	1 × 10^−6^	m
Internal friction coefficient, ξ	1 × 10^−11^	

**Table 2:** Geometry parameters of the cylindrical resonator.

Component	Value	Units
Radius of the guide ring, *R*_1_	12.3	mm
Radius of the resonant ring, *R*_2_	12.6	mm
Height of the resonant ring, *L*	5.7	mm
Height of the guide ring, *l*	3.1	mm
Thickness of the resonant ring, *h_L_*	1.2	mm
Thickness of the guide ring, *h_l_*	0.5	mm
Thickness of the bottom plate, *h_b_*	0.8	mm
